# Molecular epidemiology, virulence determinants and antimicrobial resistance of *Campylobacter* spreading in retail chicken meat in Central China

**DOI:** 10.1186/s13099-016-0132-2

**Published:** 2016-10-26

**Authors:** Tengfei Zhang, Qingping Luo, Yiluo Chen, Tingting Li, Guoyuan Wen, Rongrong Zhang, Ling Luo, Qin Lu, Diyun Ai, Hongcai Wang, Huabin Shao

**Affiliations:** 1 Hubei Key Laboratory of Animal Embryo and Molecular Breeding, Institute of Animal Husbandry and Veterinary, Hubei Academy of Agricultural Sciences, Wuhan, 430064 China; 2 College of Animal Science, Yangtze University, Jingzhou, 434025 China; 3 Hubei Animal Disease Prevention and Control Center, Wuhan, 430070 China

**Keywords:** *Campylobacter*, Chicken meat, MLST, Antimicrobial resistance, Virulence-associated genes

## Abstract

**Background:**

*Campylobacter* species are the major food-borne pathogens which could cause bacterial gastroenteritis in humans. Contaminated chicken products have been recognized as the primary vehicles of *Campylobacter* transmission to human beings. In this study, the prevalence of *Campylobacter* in retail chicken meat in Central China was investigated, and the isolates were further characterized using molecular approaches and tested for antibiotic resistance.

**Results:**

A total of 302 chicken samples purchased from April 2014 to April 2015 were tested. The level of *Campylobacter* contamination was enumerated by most probable number-PCR (MPN-PCR). The *Campylobacter* positive rate was 17.2% (52/302), with bacterial count varying from 3.6 to 360 MPN/g in positive samples. A total of 52 *Campylobacter* strains, including 40 *Campylobacter jejuni* and 12 *Campylobacter coli*, were isolated from the positive samples. To examine the genetic diversity of the isolates, multilocus sequence typing (MLST) technology was applied, which identified 23 sequence types (STs) belonging to seven clonal complexes (CCs) and unassigned. Among them, the dominant CCs of *C. jejuni* included CC-353 and CC-464, and the dominant CCs of *C. coli* were CC-828 and CC-1150. Antibiotic resistance analysis showed that all of the isolates were resistant to norfloxacin and ciprofloxacin. 23 virulence-associated genes were tested in the isolates, which showed that the number of virulence-associated genes detected in the *C. jejuni* isolates ranged from 16 to 21, while in most of the *C. coli* isolates ranged from 12 to 16. Virulence-associated genes, *flaA*, *flgB*, *flgE2*, *fliM*, *fliY* and *cadF* were detected in all isolates. V*irB11*, however, was not detected in any of the isolates.

**Conclusions:**

To the best of our knowledge, this is the first report on the contamination level and molecular biological features of *Campylobacter* strains in retail chicken meat in Central China, which showed high genetic diversity and remarkable antibiotic resistance. This study provided scientific data for the risk assessment and evaluation of *Campylobacter* contamination in retail chicken products.

**Electronic supplementary material:**

The online version of this article (doi:10.1186/s13099-016-0132-2) contains supplementary material, which is available to authorized users.

## Background

Thermophilic *Campylobacter* is the major food-borne pathogen that cause human bacterial gastroenteritis in both developed and developing countries [1]. Every year, approximately 1% of the human population in Europe are infected with *Campylobacter* [2], and the infection rate in the United States is equally high [3]. In North China in 2007, 36 cases of Guillain–Barre syndrome, which was triggered by *Campylobacter jejuni* infection, have been reported [4]. In addition, due to the prophylactic or therapeutic application of antimicrobials in animal husbandry, *Campylobacter* isolates have raised great concerns because of the spreading of the fluoroquinolone, erythromycin, and/or other drug-resistant strains [5], which limits treatment alternatives.

*Campylobacter*, mainly include *C. jejuni* and *Campylobacter coli*, are widely colonized in the intestinal tract of wild and domesticated animals and birds [6–8], even in water [9]. Chicken is one of the most popular animal-based foods worldwide, but is also an important reservoir of *Campylobacter*. The contaminated chicken products are recognized as the main source of infection [10], which highlights its potential public health threat. Several epidemiologic studies on *Campylobacter* have been carried out in parts of China. From 2008 to 2014, Wang et al. isolated large amounts of *Campylobacter* in chicken in five provinces of China, with positive rate of 18.1% for *C. jejuni* and 19.0% for *C*. *coli* [11]. Zhang et al. analyzed the genetic diversity of the *C. jejuni* isolates in Eastern China by multilocus sequence typing (MLST) and defined 94 sequence types (STs) belonging to 18 clonal complexes (CCs) [12]. To my best knowledge, few data were reported on the prevalence and contamination level of *Campylobacter* in chicken products in Central China, so the risk assessments related to food safety are also hampered by the lack of basic data.

At the same time, a number of putative virulence and toxin genes have been identified using the molecular biology methods. However, virulence mechanisms in campylobacteriosis are not fully understood. Bacterial flagellum is one of the most important virulence factors, which is associated with motility, adhesion and invasion. Konkel et al. showed that flagellar mutants had significantly reduced invasion ability [13–15]. CheY is a response regulator needed for flagellar rotation [16]. CiaB is a *Campylobacter*-invasive antigen, which is secreted through the flagellar export apparatus [13]. Some other adhesion-associated proteins have also been identified, including CadF and PEB1 [17, 18]. Several toxins were also identified in *Campylobacter*, among them, cytolethal distending toxin (CDT), composed of three subunits, has been found to be lethal for host enterocytes [19]. In addition, *virB11* gene encoded in a plasmid is a marker potentially associated with the virulence of *Campylobacter* species [20].

Retail broiler chicken meat is the last part of a broiler production chain. Therefore, the prevalence of *Campylobacter* in retail chicken meat is a clear reflection of consumer exposure. In this study, the prevalence of *Campylobacter* in retail chicken meat in Central China was investigated, and then the *Campylobacter* strains were isolated and characterized to assess their genetic relation, potential virulence factors and antibiotic resistance profiles.

## Methods

### Sampling and MPN-PCR analysis

A total of 302 samples including frozen chicken meat (n = 130) and fresh chicken meat (n = 172) were purchased from 20 supermarkets and wet markets every 3 months from April 2014 to April 2015. Each sample was homogenized, and the number of *Campylobacter* in 10 g sample homogenate was enumerated using a three-tube MPN combining with PCR method [21]. In brief, a ten-fold serial dilution series of each homogenates were prepared. Then 1 ml of each original homogenate or the diluted homogenate was transferred into each of the three tubes containing 9 ml of Bolton Enrichment Broth (OXOID, Basingstoke, England) and incubated at 42 °C for 48 h under microaerophilic condition. After incubation, total bacterial DNA was extracted and PCR amplification of *16s rDNA* was performed to detect *Campylobacter* positive tubes. For statistical analysis, the differences in frequencies were analyzed by Chi square test.

### Isolation of *Campylobacter*

*Campylobacter* strains were isolated from the positive samples and further confirmed by PCR test as previously described [22]. Isolation of the strains was performed in accordance with the International Standards Organization [ISO] 10272-1 (2006) guidelines [23].

### Antimicrobial susceptibility testing

*Campylobacter* isolates were tested for susceptibility to antimicrobial drugs using a disk diffusion assay as described previously [24], with modifications. In brief, subcultures of isolates were resuspended in Mueller–Hinton broth (OXOID, Basingstoke, UK) to obtain a turbidity equivalent to a 1.0 McFarland standard, and the suspensions were spread onto Mueller–Hinton II agar supplemented with 5% sheep blood. The disks containing each antibiotic were placed on the surfaces of the inoculated Mueller–Hinton II agar plates. These antimicrobial disks (OXOID, Basingstoke, UK) included ampicillin (Amp 10 μg), cefoperazone (Cef 75 μg), streptomycin (Str 10 μg), amikacin (Ami 30 μg), tetracycline (Tet 10 μg), sulfamethoxazole (Sul 300 μg), ciprofloxacin (Cip 5 μg), norfloxacin (Nor 10 μg), clindamycin (Cli 10 μg) and erythromycin (Ery 10 μg). Inoculated plates were incubated at 37 °C for 24–48 h in a microaerobic environment. Diameters of the inhibition zone were measured and interpreted following the disk manufacturer’s instructions and compared against the Clinical and Laboratory Standards Institute standard guidelines for aerobic gram-negative bacilli to interpret the results as susceptible, intermediate, or resistant. *E. coli* ATCC 25922 strain was included in the test for quality control.

### MLST analysis

Genomic DNA was extracted using MiniBEST Universal Genomic DNA Extraction Kit (TaKaRa, Dalian, China) according to the manufacturer’s instructions. Seven housekeeping genes, *aspA*, *glnA*, *gltA*, *glyA*, *pgm*, *tkt* and *uncA*, were amplified and sequenced based on the MLST protocol described by Dingle et al. [25]. The obtained sequences were analyzed using *Campylobacter* MLST database (http://pubmlst.org/campylobacter), and the allele numbers, sequence types (STs) and clonal complexes (CCs) were assigned. Based on the seven housekeeping gene sequences, consensus tree was constructed by using the UPGMA cluster analysis.

### Detection of virulence-associated genes

Twenty three virulence-associated genes were detected by PCR tests. The primers and amplification conditions were used as previously described [26, 27]. PCR was performed in a GeneAmp PCR System 9700 (Applied Biosystems, Darmstadt, Germany). The PCR products were subject to agarose gel electrophoresis. The DNA bands were stained with ethidium bromide and visualized using a GelDoc XR System (Bio-Rad, Shanghai, China).

## Results

### Contamination of *Campylobacter* in chicken meat

The presence of *Campylobacter* in the chicken meat was shown in Table 1. A total of 52 *Campylobacter* positives were found in the 302 collected samples of chicken meat and the contamination rate of *Campylobacter* was 17.2% in all tested samples. Hereinto, the *Campylobacter* positive rate in fresh chicken meat (22.1%) was higher than in frozen chicken meat (10.8%) in our study (*p* < 0.01). On average, 45.1 MPN/g of *Campylobacter* was detected in the positive samples, and no significant difference in contamination level was found between fresh and frozen chicken meat samples (*p* = 0.208). *Campylobacter* strains were isolated from the positive samples and species were further identified by biochemical identification and PCR tests as previously described [22]. A total of 52 *Campylobacter* strains were isolated, including 40 *C. jejuni* and 12 *C. coli*.

**Table 1:** Incidence and numbers of total *Campylobacter* in chicken meat

Source	Number of samples tested	Number of samples positive (%)	Number of samples containing total *C. jejuni* in MPN/g		
			<10^1^	10^1^–10^2^	10^2^–10^3^
Frozen chicken meat	130	14 (10.8)	2	12	0
Fresh chicken meat	172	38 (22.1)	16	20	2
In total	302	52 (17.2)	18	32	2

### Antimicrobial susceptibility

**Fig. 1:**
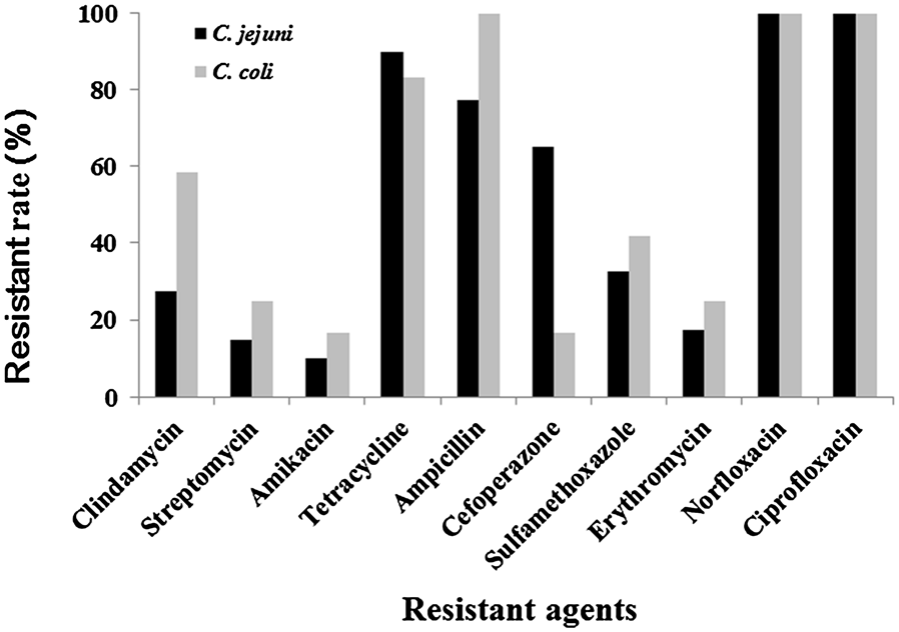
Drug resistance rates of *C. jejuni* and *C. coli* isolates

As show in Fig. 1, all the *C. jejuni* and *C. coli* isolates were resistant to norfloxacin and ciprofloxacin (100% in *C. jejuni* and *C. coli*), followed by resistance to tetracycline (90% in *C. jejuni* and 83.3% in *C. coli*) and ampicillin (82.5% in *C. jejuni* and 100% in *C. coli*). Only four *C. jejuni* (10%) and two *C. coli* (16.7%) isolates were resistant to amikacin, showing the lowest resistance rate (11.5% in total) in this study. In total, 24 antimicrobial resistance profiles were identified among 52 *Campylobacter* isolates, and all the isolates were resistant to at least three tested antimicrobial agents (Table 2). The most frequent multidrug resistance pattern was resistant to tetracycline, ampicillin, ciprofloxacin and norfloxacin. Four isolates showed resistance to nine of ten tested antimicrobial agents. The results of drug resistance test have been showed in Additional file 1: Table S1.

**Table 2:** Antimicrobial resistance patterns of *Campylobacter* isolates

No. of resistant agents	Antimicrobial resistance profile	No. of isolates	Rate (%)
3	Tet Cip Nor	3	5.8
3	Sul Cip Nor	1	1.9
3	Cef Cip Nor	1	1.9
4	Cli Amp Cip Nor	1	1.9
4	Tet Amp Cip Nor	10	19.2
4	Amp Cef Cip Nor	2	3.8
4	Tet Cef Cip Nor	2	3.8
5	Cli Amp Ery Cip Nor	1	1.9
5	Tet Amp Cef Cip Nor	7	13.5
5	Tet Amp Sul Cip Nor	1	1.9
5	Tet Cef Sul Cip Nor	1	1.9
5	Tet Cef Sul Cip Nor	1	1.9
6	Cli Tet Amp Cef Cip Nor	3	5.8
6	Str Tet Amp Cef Cip Nor	1	1.9
6	Tet Amp Cef Sul Cip Nor	2	3.8
6	Tet Amp Sul Ery Cip Nor	1	1.9
7	Cli Tet Amp Cef Sul Cip Nor	4	7.7
7	Cli Tet Amp Cef Ery Cip Nor	1	1.9
7	Tet Amp Cef Sul Ery Cip Nor	1	1.9
8	Cli Str Ami Tet Amp Ery Cip Nor	2	3.8
8	Cli Str Tet Amp Cef Sul Cip Nor	1	1.9
8	Cli Str Tet Amp Sul Ery Cip Nor	1	1.9
9	Cli Str Ami Tet Amp Cef Sul Cip Nor	1	1.9
9	Cli Str Ami Tet Amp Sul Ery Cip Nor	3	5.8

### Diversity of *Campylobacter* MLST genotype

**Fig. 2:**
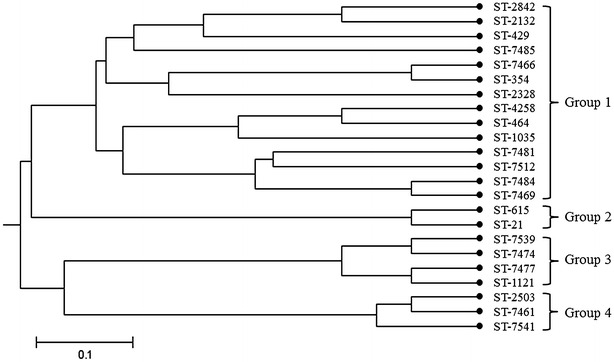
Genetic relationships of the isolates based on MLST. The consensus tree was developed by using the UPGMA cluster analysis

As shown in Table 3, 52 isolates contained a total of 23 different STs belonging to seven CCs and unassigned. Three STs including 15 isolates belonged to CC-464, accounting for 28.8% (15/52) of all isolates in this study. Nine strains belonged to ST-464, which is the most identified sequence type. The major clonal complexes also include CC-353 and CC-1150. All identified STs were further analyzed using the UPGMA cluster analysis (Fig. 2). 23 identified STs were classified into four clonal groups. All of the *C. jejuni* isolates belonged to Group 1 and 2, and all of the *C. coli* isolates belonged to Group 3 and 4. Group 1 had the largest number of STs, containing 37 strains belonging to 14 different STs (total of 71.2% isolates). Group 2, Group 3 and Group 4 included 2 STs belonging to CC-21, 4 STs belonging to CC-1150 and 3 STs belonging to CC-828 respectively.

**Table 3:** Distribution of multilocus sequence types and virulence associated factors in *C. jejuni* and *C. coli* isolates

Species	Group	CCs	STs	No.^a^	*flaA*	*flab*	*flhA*	*flhB*	*flgB*	*FlgE2*	*fliM*	*fliY*	*CiaB*	*iamA*	*VirB11*	*CadF*
*C. jejuni*	1	48	429	2	+	+	+	+	+	+	+	+	+	+	−	+
		353	2132	2	+	+	+	+	+	+	+	+	+	+	−	+
			2842	3	+	2/3^b^	+	+	+	+	+	+	2/3	+	−	+
			7512	2	+	+	+	+	+	+	+	+	+	+	−	+
		354	354	3	+	+	+	+	+	+	+	+	2/3	+	−	+
			7466	1	+	+	+	+	+	+	+	+	−	+	−	+
		464	464	9	+	+	+	+	+	+	+	+	+	+	−	+
			7469	5	+	+	+	+	+	+	+	+	+	+	−	+
			7484	1	+	+	+	+	+	+	+	+	+	+	−	+
		UA	1035	3	+	+	+	+	+	+	+	+	+	−	−	+
			2328	3	+	+	+	+	+	+	+	+	2/3	+	−	+
			4258	1	+	+	+	+	+	+	+	+	+	+	−	+
			7481	1	+	+	+	+	+	+	+	+	+	+	−	+
			7485	1	+	+	+	+	+	+	+	+	+	+	−	+
	2	21	21	2	+	+	+	+	+	+	+	+	+	+	−	+
			615	1	+	+	+	+	+	+	+	+	+	+	−	+
*C. coli*	3	1150	1121	1	+	+	+	+	+	+	+	+	−	−	−	+
			7539	1	+	+	+	−	+	+	+	+	+	+	−	+
			7474	5	+	+	4/5	1/5	+	+	+	+	−	1/5	−	+
			7477	1	+	+	−	−	+	+	+	+	−	−	−	+
	4	828	2503	1	+	+	+	+	+	+	+	+	−	−	−	+
			7461	1	+	+	−	+	+	+	+	+	−	+	−	+
			7541	2	+	+	+	+	+	+	+	+	1/2	1/2	−	+
Total				52	52	51	49	46	52	52	52	52	38	4	0	52

Species	Group	CC	ST	No.	*docA*	*docB*	*docC*	*cdtA*	*cdtB*	*cdtC*	*wlaN*	*cgtB*	*cheY*	*ilpA*	*kpsM*	
*C. jejuni*	1	48	429	2	+	+	+	+	+	+	−	−	+	+	+	
		353	2132	2	+	+	−	+	+	+	−	−	+	+	−	
			2842	3	+	+	1/3	+	+	+	2/3	−	+	+	1/3	
			7512	2	+	+	−	+	+	+	−	−	+	+	−	
		354	354	3	+	+	1/3	+	+	+	−	−	+	+	2/3	
			7466	1	+	+	−	+	+	+	−	−	+	+	−	
		464	464	9	+	+	7/9	+	+	+	−	−	+	+	+	
			7469	5	+	+	−	+	+	+	−	−	+	+	+	
			7484	1	+	+	−	+	+	+	−	−	+	+	+	
		UA	1035	3	+	+	+	+	+	+	−	−	+	+	−	
			2328	3	+	+	−	+	+	+	1/3	−	+	+	+	
			4258	1	+	+	−	+	+	+	−	−	+	+	+	
			7481	1	+	+	−	+	+	+	+	−	+	+	+	
			7485	1	+	+	−	+	+	+	−	+	+	+	+	
	2	21	21	2	+	+	+	+	+	+	−	+	+	+	+	
			615	1	+	+	−	+	+	+	−	+	+	+	+	
*C. coli*	3	1150	1121	1	−	−	−	−	+	+	−	−	+	+	+	
			7539	1	+	−	−	+	+	+	−	−	−	+	+	
			7474	5	−	−	−	+	3/5	4/5	−	−	4/5	+	+	
			7477	1	−	−	−	+	+	+	−	−	+	+	+	
	4	828	2503	1	−	−	−	+	+	+	−	−	+	+	−	
			7461	1	−	−	−	+	+	+	−	−	−	+	−	
			7541	2	1/2	1/2	−	+	+	1/2	−	−	+	1/2	−	
Total				52	42	41	16	51	50	50	4	4	49	51	39	

“+” means present in all isolates, “−” means absent in all isolates

^a^Numbers of strains belong to each sequence type

^b^Present in two of three isolates

### Distribution of virulence-associated genes

**Fig. 3:**
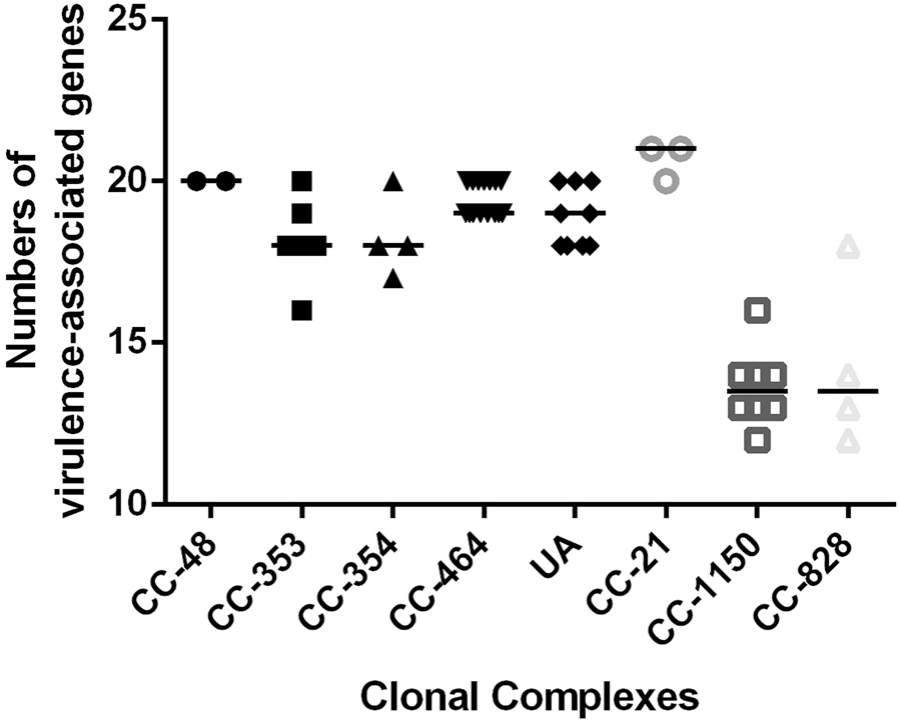
The number of virulence associated genes in each CCs of tested *Campylobacter* isolates

A total of 23 virulence-associated genes were screened by PCR in this study (Table 3). *FlaA*, *flgB*, *flgE2*, *fliM*, *fliY* and *cadF* were detected in all *Campylobacter* isolates, while *virB11* was not detected in any isolates. Various detection rates were observed for the rest of the virulence-associated genes. Among them, *flaB* (51/52, 98.1%), *cdtA* (51/52, 98.1%), *cdtB* (50/52, 96.2%), *cdtC* (50/52, 96.2%), *ilpA* (51/52, 98.1%), *cheY* (49/52, 94.2%) and *flhA* (49/52, 94.2%) were found in more than 90% isolated strains. In contrast, *wlaN* (7.7%, 4/52) and *cgtB* (7.7%, 4/52) were only detected in four strains, respectively. Strains with all tested virulence genes were not evenly distributed among *C. jejuni* and *C. coli* isolates (Fig. 3). The number of virulence-associated genes detected in *C. jejuni* (in Group 1 and Group 2) ranged from 16 to 21. Two strains belonged to CC-21 contained the most virulence-associated genes (n = 21). In contrast, less virulence-associated genes, ranging from 12 to 16, were detected in most of the *C. coli* isolates (in Group 3 and Group 4), except one strain belonging to CC-828 contained 18. Two strains with the fewest virulence-associated genes (n = 12) were in CC-1150 and CC-828 respectively.

## Discussion

Chicken and their products are commonly consumed by human, but little detailed information is available about *Campylobacter* from retail chicken meat in China. In this study, we found that 17.2% of the retail chicken meat samples were contaminated with *Campylobacter* and the contamination levels ranged from 3.6 to 360 MPN/g in positive samples in Central China. Furthermore, the major MLST genotypes of *Campylobacter* were CC-353, CC-464 and CC-1150, meanwhile all the isolates were fluoroquinolone-resistant. To my best known, this is the first surveillance report of *Campylobacter* enumeration study of retail chicken meat in Central China.

Wong et al. study showed that the prevalence of *C. jejuni* and *C. coli* was as high as 89.1% in chicken meat with a total bacterial count varying from 0 to 110 MPN/g in New Zealand [28]. In Beijing, China, 26.3% of the retail whole chicken carcasses were contaminated by *Campylobacter* [29]. Our data showed that, the contamination rate of *Campylobacter* was lower than some of the developed countries. The relatively low positive rate of *Campylobacter* was also reported in East China [30]. A risk assessment revealed that in an outbreak of *C. jejuni* infection, the infection rate and ingestion dose were 37.5% and 360 MPN [31]. In our study, the numbers of contaminated bacteria in 65.4% (34/52) of *Campylobacter* positive samples were more than 10 MPN/g, in other words, more than 10^4^ MPN/kg. The high contamination levels of *Campylobacter* suggested that the buyers should take care of the food processing process [32].

It is reported that antibiotics resistant strains of *Campylobacter* lead to more severe disease in humans [33]. High resistance rates were observed in our study. It is certain that the severe multi-drug resistance increases the threat to public safety. Fluoroquinolones and tetracycline are used as therapeutic drugs in severe cases of infection frequently [34]. However, all of the isolates were fluoroquinolone-resistance, while the tetracycline-resistance reached 88.5%. High fluoroquinolone and tetracycline-resistance rates were also reported in other studies in China and other countries [27, 29]. In contrast, in countries with strict antimicrobial controls, much lower resistance rates of ciprofloxacin were observed in *Campylobacter* [35, 36]. Erythromycin is the preferred drug for treatment of human campylobacteriosis in lots of countries [34]. The resistance rate to erythromycin was 19.2%, which was lower than most of the tested drugs in our study. Lower resistance rate to erythromycin was also reported in other countries [27, 36]. In our study, most of the isolates (82.7%) were sensitive to amikacin and streptomycin, and similar results were reported in several previous studies [37, 38].

Our study revealed a high diversity of genotypes among 52 *Campylobacter* isolates obtained from the supermarkets and wet markets in Central China, including CC-353, CC-464, CC-1150 and so on. It is noteworthy that all of the unassigned STs are clustering in Group one and most of them are clustering with CC-353 or CC-464, which suggesting their close genetic relationship with the dominant clonal complexes in Central China. CC-353 and CC-354 are also the most frequently reported *C. jejuni* genotypes in human disease, such as in Greece and Scotland [39, 40]. In retail chicken carcasses in Beijing, North China, the dominant clonal complexes of *C. coli* were CC-828 and CC-1150, which were the same as in our study, but the clonal complexes of *C. jejuni* were diverse [29]. In Zeng’s study, ST-21 was the major type in East China, accounting for 39.3% of the total strains [30]. In our study, however, only two *C. jejuni* strains belonging to ST-21 were isolated. These results suggested that the dominant clonal complexes of *C*. *jejuni* were discrepant in different regions of China, but the dominant clonal complexes of *C. coli* were similar. In our previous epizootic investigation of some chicken farm in Central China, we found that the positive rate of *Campylobacter* in cloacal swabs was 15.8%. Within seven observed CCs in this study, six CCs except CC-48 were also observed (unpublished), which revealed the high similarity between isolates from farms and markets. Initial meat contamination with *Campylobacter* may come from the destructive chicken intestine during processing [41]. This study provided supporting evidence and further indicated the importance of good biosecurity during the manufacturing process, especially for ensuring the integrity of intestine. Otherwise, in order to reduce *Campylobacter* contamination in chicken meat, we think the most important thing is to lower the bacterial count in chickens. Incorporating antibiotics into feed might help reduce the levels of colonization, but will produce resistant strains. Rational use of environment-friendly microbial feed additive seems to be a good way.

Potential virulence properties include motility, chemotaxis, colonization, adhesion and invasion of epithelial cell, intracellular survival, and formation of toxins. To understand better the virulence potential of our isolates, we characterized 23 virulence-associated genes in these processes [26, 42]. Flagellar is one of the most important factors associated with adhesion, invasion and colonization. High detection rates of flagellar genes were observed in both *C. jejuni* and *C. coli*. Among them, five genes (*flaA*, *flgB*, *flgE2*, *fliM* and *fliY*) were detected in all tested strains and another three (*flaB*, *flhA* and *flhB*) were detected in more than 88% of the strains. High detection rates of flagellar genes have been also reported in other studies [26, 43], except *flab* which was absent in 8 of 17 tested *C. jejuni* in Koolman’ study [44]. In contrast, *flaB* was only absent in one of our tested *C. jejuni* strains (1/40). A fibronectin-binding protein encoded by *cadF* was another virulence factor detected in all strains. *wlaN* and *cgtB* genes were detected in a few strains, in which their detection rates were both 7.7% (4/52). The low detection rate of these two genes may because that they are not essential for colonization and pathogenesis of *Campylobacter*. *virB11* is located in the pVir plasmid [45]. We could not detect *virB11*, indicating that all of our isolates did not have the pVir plasmid.

Few studies reported the distribution of virulence factors in *C. coli*. As shown in Fig. 3, we found that the distribution of virulence-associated factors in *C. jejuni* and *C. coli* were different. The results showed that the number of virulence-associated genes detected in *C. jejuni* isolates ranged from 16 to 21, while in most of the *C. coli* isolates, ranging from 12 to 16. Especially for invasion related genes, *ciaB* and *iamA*, and chemotaxis factors, *docA* and *docB*, the detection rates of these four genes in *C. jejuni* were much higher than in *C. coli* (*ciaB*, 90 vs 16.7%; *iamA*, 92.5 vs 33.3; *docA*, 100 vs 16.7%; *docB*, 100 vs 8.3%). In addition, chemotaxis factors *cheY* was present in all *C. jejuni* strains but was absent in 3 of the 12 *C. coli* strains. Some subunit of cytolethal distending toxins were also absent in a small part of *C. coli*. As reported, most of campylobacteriosis were caused by *C. jejuni* [46]. It may because that the prevalent strains of *C. jejuni* contained more virulence factors than *C. coli*. In this study, most virulence associated genes were found in two *C. jejuni* isolates belonging to ST-21. CC-21 shows a large overlap in genetic variation among reservoirs, including both animals (e.g. cattle, sheep, pig, wild bird) and environmental sources, more virulence associated genes may contribute to its adaptation to a variety of environment [47, 48]. Although CC-353 and CC-354 are frequently reported in human disease, the numbers of virulence associated genes were not more than others. We inferred that some of the detected virulence associated genes might not be essential for human infection. We think these results will provide useful information for further understanding the mechanisms of pathogenesis in *C. jejuni* and *C. coli*.

## Conclusions

This study firstly provided the information about the contamination levels and genetic diversity of *Campylobacter* in retail chicken meat in Central China. We also showed antibiotic susceptibility profiles and distribution of virulence-associated genes. This study provided a basic data for risk assessment of food-borne transmission of *Campylobacter*. Further investigations are needed to improve our knowledge about the epidemiology of *Campylobacter* in human in Central China.

## Additional file

**Additional file 1.** **Table S1**. The results of the durg resistance testing of each strain.

## Abbreviations

MPN: most probable number
MLST: multilocus sequence typing
STs: sequence types
CCs: clonal complexes
